# Characterization of strains of *Neisseria meningitidis* causing meningococcal meningitis in Mozambique, 2014: Implications for vaccination against meningococcal meningitis

**DOI:** 10.1371/journal.pone.0197390

**Published:** 2018-08-08

**Authors:** Alcides Moniz Munguambe, António Eugénio Castro Cardoso de Almeida, Aquino Albino Nhantumbo, Charlotte Elizabeth Come, Tomás Francisco Zimba, José Paulo Langa, Ivano de Filippis, Eduardo Samo Gudo

**Affiliations:** 1 Microbiology Laboratory, National Institute of Health, Ministry of Health, Maputo, Mozambique; 2 Laboratory of Reference Microorganisms, National Institute of Quality Control of Health (INCQS); Oswaldo Cruz Foundation (FIOCRUZ), Rio de Janeiro, Brazil; 3 Maputo Central Hospital, Maputo, Mozambique; 4 National Institute of Health, Ministry of Health, Maputo, Mozambique; Defense Threat Reduction Agency, UNITED STATES

## Abstract

**Introduction:**

In sub Saharan Africa, the epidemiology, including the distribution of serogroups of strains of *N*. *meningitidis* is poorly investigated in countries outside “the meningitis belt”. This study was conducted with the aim to determine the distribution of serogroups of strains of *N*. *meningitidis* causing meningococcal meningitis in children and adults in Mozambique.

**Methods:**

A total of 106 PCR confirmed *Neisseria meningitidis* Cerebrospinal Fluid (CSF) samples or isolates were obtained from the biobank of acute bacterial meningitis (ABM) surveillance being implemented by the National Institute of Health, at three central hospitals in Mozambique, from January to December 2014. Serogroups of *N*. *meningitidis* were determined using conventional PCR, targeting *siaD* gene for *Neisseria meningitidis*. Outer Membrane Proteins (OMP) Genotyping was performed by amplifying *porA* gene in nine samples.

**Results:**

Of the 106 PCR confirmed *Neisseria meningitidis* samples, the most frequent serotype was A (50.0%, 53/106), followed by W/Y (18.9%, 20/106), C (8.5%, 9/106), X (7.5%, 8/106) and B (0.9%, 1/106). We found non-groupable strains in a total of 15 (14.2%) samples. *PorA* genotypes from nine strains showed expected patterns with the exception of two serogroup C strains with P1.19,15,36 and P1.19–36,15 and one serogroup X with P1.19,15,36, variants frequently associated to serogroup B.

**Conclusion:**

Our data shows that the number of cases of meningococcal meningitis routinely reported in central hospitals in Mozambique is significant and the most dominant serogroup is A. In conclusion, although serogroup A has almost been eliminated from the “meningitis belt”, this serogroup remains a major concern in countries outside the belt such as Mozambique.

## Introduction

*Neisseria meningitidis*, a Gram-negative diplococcus is one of the major etiologies of meningitis and septicemia [1]. Meningococcal meningitis is often serious and potentially fatal and death occurs on average in 10% of untreated patients [2]. According to the World Health Organization (WHO), an estimated 500,000 cases and 50,000 deaths are attributed to *N*. *meningitidis* each year worldwide [2]. Survivors often suffer of severe sequels [3, 4]. Children and young adults are the main victims [4, 5].

*N*. *meningitidis* is divided into 13 serogroups based on the antigenicity of the polysaccharide capsule, of which, the serogroups A, B, C, W, Y, and X cause life-threatening invasive disease and are most frequently implicated in epidemics or outbreaks of meningococcal meningitis [4, 6].

Worldwide, the highest incidence and burden of meningococcal meningitis is reported in sub-Saharan African, in a region known as the meningitis belt, which traditionally comprises 26 countries from Senegal in the west to Ethiopia in the east [2, 7]. In this region, the incidence of meningococcal meningitis is very high and can reach up to 1,000 cases/100,000 inhabitants during epidemics [4, 8]. In the “meningitis belt”, the disease has been extensively studied and serogroup A was historically the dominant serogroup causing meningococcal meningitis before the introduction of conjugated vaccination [4, 7, 9]. Vaccination against meningococcus A (MenA), using conjugated vaccine was progressively introduced in several countries in the “meningitis belt” starting in 2010 and led to a significant drop in the incidence of meningococcal meningitis [8, 10, 11]. However, outside the “meningitis belt”, the meningococcal vaccine is underutilized.

In contrast, in Mozambique and most of the African countries located outside the “meningitis belt”, the epidemiology of meningococcal meningitis remains widely unknown, because until recently, *N*. *meningitidis* had been regarded as a minor cause of meningitis and consequently remained ignored in these countries.

In recent years, public health relevance of meningococcus is increasing in Mozambique and other countries outside the “meningitis belt” in sub Saharan Africa, for the following reasons: i) bacterial meningitis caused by *H*. *influenzae* type b and *S*. *pneumoniae* declined in the country after the introduction of vaccination [12–14] and consequently, *N*. *meningitidis* is gradually becoming a leading cause of Acute Bacterial Meningitis (ABM), ii) high vulnerability of the country to extreme climate events such as droughts, is known to enhance damage of nasopharyngeal mucosal, thus increasing the risk of meningococcal meningitis [5, 15–18], iii) socio demographics changes, such as, increase of international travel, rapid and unplanned urbanization, together with poor sanitation and overcrowding. All of these changes are known to enhance the spread of *N*. *meningitidis* [2, 5, 19] and there is fear that meningitis is expanding to other countries not previously considered for surveillance of *N*. *meningitidis* [8, 20]. In this regard, there is an urgent need for data on the burden of meningococcal meningitis in countries outside the “meningitis belt”.

In regard to Mozambique, few data exist on the epidemiology of meningococcal meningitis. Two studies conducted in Manhiça district, a small rural district situated in the southern region of the country, found that the incidence rate of meningococcal meningitis was higher than expected for a country outside the “meningitis belt” [21, 22]. Another study conducted by Zimba and collaborators, in 2009, found that N. meningitides was the most common cause of community acquired meningitis in patients admitted to Maputo Central Hospital between August 2007 and March 2008 [23]. Notably, the study conducted in Manhiça district, was the only that investigated the distribution of serogroups of *N*. *meningitidis* in Mozambique and was conducted more than 8 years ago in a rural district in southern Mozambique and for this reason it is difficult to extrapolate to other regions of the country, due to the geographical heterogeneity of distribution of serogroups *N*. *meningitidis*. No data is available for the rest of the country. In this context, we conducted this study with the aim to determine the distribution of serogroups of meningococcus among *Neisseria meningitidis* positive CSF samples or isolates from three central Hospitals situated in major geographical regions in Mozambique. Knowledge of the serogroup distribution of strains causing meningococcal meningitis in Mozambique is important to current and ongoing discussions on the potential use of the meningococcal vaccine in the country.

## Methods

### Study design and setting

**Fig 1:**
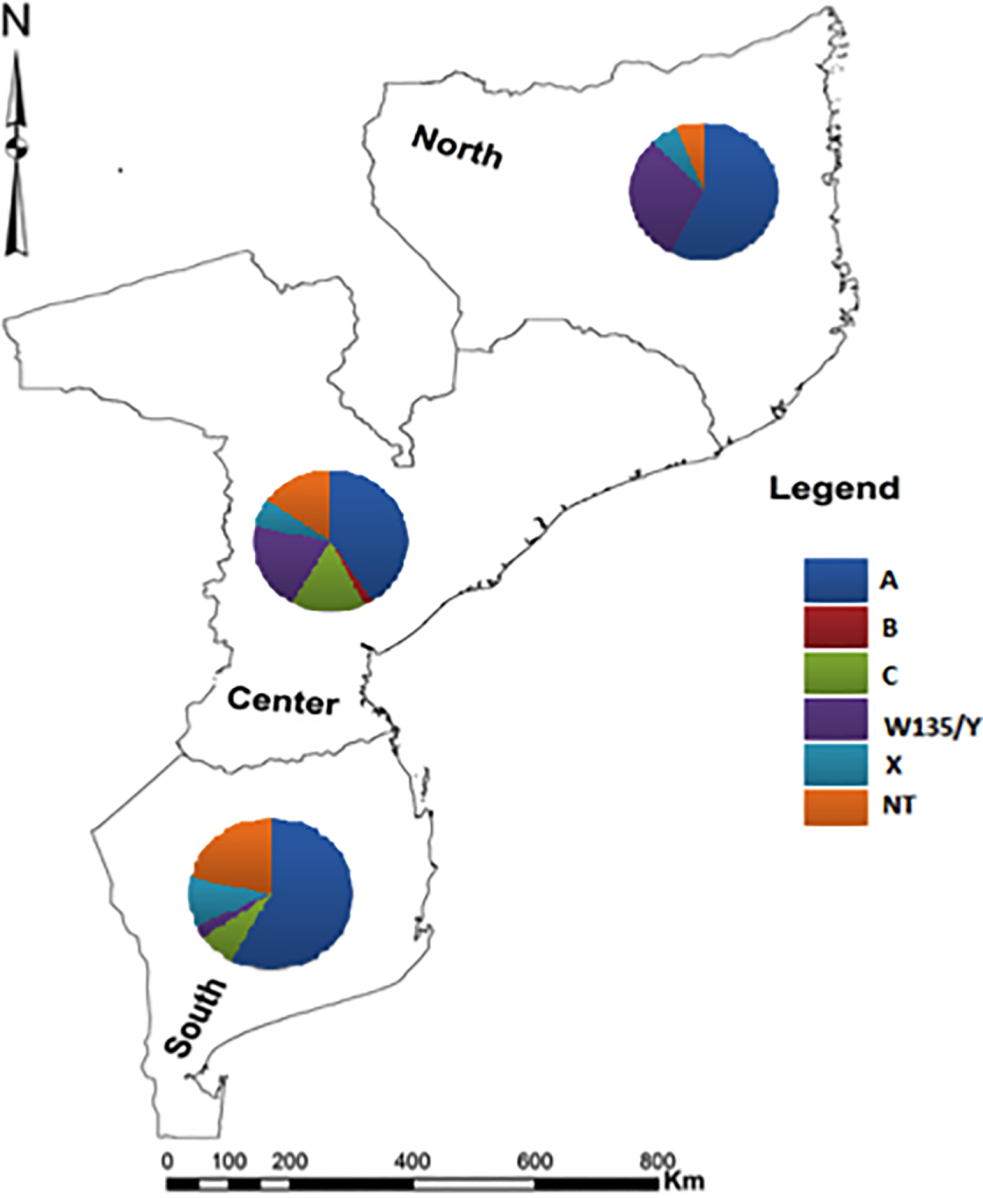
Distribution of *Neisseria meningitidis* serotypes in Mozambique.

A cross-sectional study was conducted to determine the serogroup of all PCR confirmed *N*. *meningitidis* Cerebrospinal Fluid (CSF) samples or isolates (n = 106) stored in the biobank of the Microbiology Reference Laboratory (MRL), at the National Institute of Health. All samples stored in this biobank were collected as part of the ABM surveillance being implemented by the National Institute of Health at three largest hospitals in the country, namely Maputo Central Hospital, Beira Central Hospital and Nampula Central Hospital, situated in the southern, center, and northern regions of Mozambique, respectively (See Fig 1). Only *N*. *meningitidis* positive samples identified between January and December 2014 were selected for this study.

Geographical and demographics characteristics of the three hospitals are summarized in the S1 Table and are based on the Population Census conducted in 2007 [24]. These three cities are characterized by presence of a small urban area surrounded by a vast and rapidly growing periurban slums characterized by poor sanitation, precarious housing, and overcrowding.

The climate in Mozambique is tropical, with two seasons: the raining season from November through April and the dry season during the rest of the year. The average temperature in Mozambique varies between 22 and 24°C and the average annual rainfall is 1200 mm. The relative air humidity is high, ranging between 70–80%.

### Ethics statement

The use of the samples and isolates stored at the biobank of the MRL at the National Institute of Health for this study was approved by the Mozambican National Bioethics Committee (reference number is: 304/CNBS/12).

### Selection of samples and isolates of *N*. *meningitidis*

We selected all *N*. *meningitidis* positive samples of isolates available at the MRL that were identified as part of sentinel surveillance for ABM being implemented by the National Institute of Health in Mozambique.

### Demographic data

Demographic data from each *N*. *meningitidis* positive patient was obtained from the ABM surveillance database at MRL.

### Laboratory testing

#### Storage conditions and shipment to Brazil

CSF samples or isolates from each *N*. *meningitidis* positive patient was preserved at -70°C at the MRL until shipment to the National Institute of Quality Control of Health in Rio de Janeiro- Brazil for molecular testing. Samples and isolates were shipped in dry ice.

#### DNA extraction and purification

Bacterial DNA extraction and purification from CSF and from isolates of *N*. *meningitidis* was performed using the DNeasy Blood & Tissue Kit (QIAGEN, QIAGEN Group, Germany) according to manufacturer’s instructions. The purified genomic DNA was collected into a 1,5ml tube and stored at -20°C.

#### Molecular identification of *N*. *meningitidis* and meningococcal serogroups

Molecular identification of *N*. *meningitidis* strains from purified DNA was performed using a conventional *nspA*-PCR as previously described [25]. After *nspA*-PCR, positive samples, were submitted to multiplex *siaD*-PCR for serogroup determination (A, B, C, W, X and Y) according to previously described protocols [26, 27] targeting the *siaD* cassette responsible for the synthesis and processing of capsular polysaccharides.

#### PorA antigen gene sequencing

From the 106 confirmed meningococcal samples, nine were submitted to *porA* gene amplification in order to determine the genetic variants of their Variable Regions (VR) according to previously described protocols [28]. *porA* gene sequences were submitted to the *Neisseria* PubMLST database (https://pubmlst.org/neisseria/) to determine *porA* variants. The remaining samples were excluded due to poor quality or insufficient amount of DNA.

## Results

### General characteristics of participants

Extracted DNA samples were submitted to *nspA*-PCR amplification as previously described [25] to confirm *N*. *meningitidis* infection. Of the 106 *nspA*-PCR confirmed CSF samples or isolates of *N*. *meningitidis* positive patients, 54 (50.9%) were female, while 45 (42.5%), were from the central region, 33 (31.1%) were from the northern region and 28 (26.4%) were from the southern region (Table 1).

**Table 1:** Frequencies of serogroups of *Neisseria meningitidis* stratified by geographical region, age and gender in 2014.

Characteristic				*Neisseria meningitidis* serogroup					
			Total	A	B	C	W/Y	X	Nm-NG
			n (%)	n (%)	n (%)	n (%)	n (%)	n (%)	n (%)
Total			106 (100%)	53 (50.0%)	1 (0.9%)	9 (8.5%)	20 (18.9%)	8 (7.5%)	15 (14.2%)
**Gender**									
	Male		52 (49.1)	26 (49.1)	0 (0.0)	5 (55.6)	11 (55.0)	3 (37.5)	8 (53.3)
** **	Female		54 (50.9)	27 (50.9)	1 (100)	4 (44.4)	9 (45.0)	5 (62.5)	7 (46.7)
**Region**			**n = 106**	**n = 53**	**n = 1**	**n = 9**	**n = 20**	**n = 8**	**n = 15**
	North		33 (31.1)	19 (35.8)	0 (0.0)	0 (0.0)	10 (50.0)	2 (25.0)	2 (13.3)
	Center		45 (42.5)	18 (34.0)	1 (100)	7 (77.8)	9 (45.0)	3 (37.5)	7 (46.7)
	South		28 (26.4)	16 (30.2)	0 (0.0)	2 (22.2)	1 (5.0)	3 (37.5)	6 (40.0)
**Age, years**			**n = 104**	**n = 52**	**n = 1**	**n = 9**	**n = 19**	**n = 7**	**n = 15**
		Median (IQR)	15 (6–32)	19 (15–36)	1 (1–1)	12 (4–25)	13 (9–30)	16 (8–32)	12 (5–23)
**Age category, years**			**n = 104**	**n = 52**	**n = 1**	**n = 9**	**n = 19**	**n = 7**	**n = 15**
	0–5		26 (25.0)	16 (30.2)	1 (100)	3 (33.3)	1 (5.3)	1 (14.3)	4 (27.7)
	6–18		31 (29.8)	10 (18.9)	0 (0.0)	3 (33.3)	10 (52.6)	3 (42.9)	5 (33.3)
	> 18		47 (45.2)	26 (50.1)	0 (0.0)	3 (33.3)	8 (42.1)	3 (42.9)	6 (40.0)

The median age of these patients was 15 years (IQR: 6–32 years). In terms of distribution by age category, 25.0% (26/104), 29.8% (31/104) and 45.2% (47/104) of *N*. *meningitidis* samples were from patients of the age category 0–5, 6–18 and >18 years old, respectively (see Table 1).

### Frequency of serogroups of *N*. *meningitidis*

The most frequent serogroup of *N*. *meningitidis* was A (50.0%, 53/106), followed by W/Y (18.9%, 20/106), C (8.5%, 9/106), X (7.5%, 8/106), while the less frequent was serogroup B (0.9%; 1/106); non-groupable (NG) strains corresponded to 14.2% (15/106) (Table 1).

Frequency of serogroup A was higher in the age group >18 years when compared with age group 6–18 years old. Only one case of serogroup B was reported in the group 0–5 years. Distribution of serogroup C, was similar in each age group (3/9; 33.3% in the age categories 0–5 years, 6–18 years and >18 years old). Serogroup W/Y, was more frequent in the age group of 6–18 years and age group >18 years as compared to the age group 0–5 years old, in which only 1 case was reported. Frequency of serogroup X was lower in the age category 0–5 years old.

Non-groupable strains showed a similar distribution among all age groups.

### Geographic distribution of serogroups of *N*. *meningitidis*

Data from Table 1 don’t show important differences in distribution of *N*. *meningitidis* serogroups A, X and NG over the three regions. The only case of serotype B, occurred in the central Mozambique.

Serogroup C was more frequent in the central region of the country (7/9, 77.8%) followed by the southern region (2/9, 22.2%). No *N*. *meningitidis* serogroup C were isolated in the north.

The northern region had the highest frequency of W/Y serogroup (10/20; 50.0%), followed by center of the country (9/20, 45.0%) and the lower frequency was reported in the southern region (1/20; 5.0%).

### OMP genotyping

*PorA* genotypes were identified from nine of the 106 *nspA*-PCR confirmed meningococcal samples (Table 2). Specific *porA* patterns are generally associated with capsular serogroups and clonal complexes. In our study, the majority of C strains, showed *porA* variants normally associated to this serogroup. However, two serogroup C strains were characterized as P1.19,15,36 and P1.19–36,15 and one serogoroup X as P1.19,15,36. These PorA genotypes are frequently associated to serogroup B strains.

**Table 2:** PorA variants distribution among serogroups.

Serogroup	Gender	Age	Date received	Source	CSF	PorA-VR1	PorA-VR2	PorA-VR3
W	M	ND	25/07/2014	Nampula	Cloudy	5	2	36–2
W	F	9	11/7/14	Beira	Clear	5–9	ND	ND
C	M	18	18/11/2014	Beira	Clear	19	15	36
C	F	36	27/11/2014	Beira	Clear	19–36	15	ND
B	F	1	1/3/15	Beira	Cloudy	19	15	36
X	F	6	1/3/15	Beira	Clear	19	15	36
A	F	4	1/3/15	Beira	Clear	5	2	36–2
C	M	ND	1/3/15	Beira	Clear	5	2	36–2
A	F	21	6/2/14	Beira	Bloody	19–36	15	ND

ND: not determined.

## Discussion

In Mozambique and other countries outside the “meningitis belt” in sub Saharan Africa, little is known about the epidemiology of *N*. *meningitidis* and only few countries outside the “meningitis belt” have introduced routine or reactive vaccination against *N*. *meningitidis*.

In this study, we identified serogroups of meningococcus in *N*. *meningitidis* positive CSF or isolates samples obtained from the biobank of stored samples at the MRL. Of note, this is the first study that investigate the distribution of serogroups of meningococcal meningitis in the three main geographical regions of the country and showed that serogroups A was the dominant serogroup of *N*. *meningitidis*. This finding is in agreement with data from the “meningitis belt” before the introduction of conjugated vaccination against serogroup A (MenAfriVac) [7, 29]. Historically this serogroup has been considered the main cause of outbreaks and epidemics in the “meningitis belt”, which dropped after introduction of vaccination [4, 10, 11]. Data from our study suggests that a potential vaccination against serogroup A of *N*. *meningitidis* would cover a significant proportion of cases *N*. *meningitidis* and could promote a serogroup shift towards non serogroup-A meningococcal meningitis similar of what occurred in the “meningitis belt” [8]. However, findings from this study are different from results of a study conducted in Manhiça district in southern Mozambique, which found that serogroup W was the most prevailing serogroup [21]. The discrepancy can be explained by the fact that, the study conducted in Manhiça district is more than 9 years old and Manhiça is a small rural district, while our study was conducted in the three largest urban areas of the country. Indeed, is well known that the epidemiology of *N*. *meningitidis* varies in different places and over time [30].

Notably, frequency of serogroup A was similar in the three regions of the country, suggesting that serogroup A is the dominant serogroup throughout the country.

The design of our study does not allow to determine incidence of *N*. *meningitidis*, however, results from studies conducted in Manhiça districts found that incidence rates of *N*. *meningitidis* were higher than expected for a country situated outside the “meningitis belt” [21, 22]. This suggest that meningococcus may be more important than assumed. Vaccination is considered the best option to prevent and control meningococcal meningitis, but no meningococcal vaccine is used in Mozambique. WHO suggests that aside from the “meningitis belt”, vaccination against MenA should be considered in countries with moderate to high incidence [31]. Taking into consideration that incidence was ranked as intermediate in Manhiça district, and Mozambique has an enhanced risk for meningococcal meningitis due to climatic vulnerability and socio demographics changes [32], vaccination against meningococcus should be considered at least for high risk groups or in response to outbreaks.

The age distribution of *N*. *meningitidis* of this study strongly suggests that meningococcal meningitis occurs in children, adolescent and young adults, and any decision about vaccination should take into account this aspect. For instance, the massive vaccination in the “meningitis belt” targeted people from 1–29 years old [4].

The second and third most frequent serogroups were W/Y (18.9%) and C (8.5%), suggesting that these serogroups have the potential to cause outbreaks. Indeed, there was a drop in the burden of serogroup A related meningitis in the “meningitis belt” after the introduction of mass vaccination using MenA conjugated vaccine formulation, serogroups W and C emerged as common etiologies of meningococcal meningitis and serogroups W subsequently become a leading cause of most recent meningococcal meningitis outbreaks [8, 10, 18, 33–36]. Serogroup X was reported in 7.5% of the samples, showing its public health importance and indeed, this serogroup is emerging in sub Saharan Africa as an important cause of meningococcal meningitis [37]. Since effective vaccines are available for serogroups A, C, W, and Y, we recommend that any discussion about potential use of meningococcal vaccination in Mozambique should consider the use of a vaccine formulation that covers multiple serogroups.

The low frequency of serogroup B (0.9%), is in agreement with results of other studies conducted in Africa [7, 33]. This serogroup is more common in other continents [4, 18]. Two serogroup C strains out of 9 (22.2%) and one serogroup X strain out of 8 (12.5%) were characterized with *porA* genotypes frequently associated to serogroup B. This feature emphasizes the need to monitor the potential spread of new clones in the region which should be followed by extended genotyping of circulating strains including *fetA* and MLST typing. This justifies an enhanced system of surveillance by molecular typing of such isolates.

We acknowledge the lack of information on the total number of children at risk necessary to calculate the incidence of *N*. *meningitidis* represents a limitation of our study. However, the number of cases reported in this study is significant and taking into account that our surveillance seriously underestimates the real burden of disease, we can anticipate that several other cases or outbreaks occurred silently in these cities.

This indicates that *N*. *meningitidis* represents a serious, but under recognized threat in the country.

We also acknowledge that due to poor quality or lack of sufficient amount of DNA, the number of samples genotyped for assessment of *porA* genotypes was small. However, despite the small number, the information is relevant for Mozambique, where data about XYZ are scarce.

## Conclusion

In conclusion, our data shows that serogroup A of *N*. *meningitidis* is the dominant serogroup causing meningococcal meningitis in Mozambique, suggesting that although it has been almost eliminated in meningitis belt, serogroup A is still a public health concern in countries outside meningitis belt such as Mozambique where vaccination against *N*. *meningitidis* has not yet been introduced. Nevertheless, serogroup W135/Y, represents the second most common etiology of meningococcal meningitis with a frequency of 18.9% and serogroup C and X together accounted for 16.0% of the tested strains. Data from this study expand the limited evidence of *N*. *meningitidis* in Sub Saharan countries outside the “meningitis belt”, and are relevant to ongoing discussions on the potential introduction of vaccination against *N*. *meningitidis* in Mozambique.

## Supporting information

S1 Table Geographical and demographics characteristics of the three hospitals. (DOCX)

S2 Table Minimal dataset. (XLS)
