# Supplementary material for: Characterization of strains of Neisseria meningitidis causing meningococcal meningitis in Mozambique, 2014: Implications for vaccination against meningococcal meningitis
Source: PLoS One. 2018 Aug 8;13(8):e0197390. doi: 10.1371/journal.pone.0197390 (PMC6082507; doi:10.1371/journal.pone.0197390)
Supplement: S1 Table — (DOCX) [file pone.0197390.s001.docx]

| **Hospital** | **Province** | **Pop (hab)** | **Surface Area (Km^2^)** | **Pop density (hab/Km^2^)** |
| --- | --- | --- | --- | --- |
| Maputo Central Hospital | Maputo City | 1,205,709 | 22,693 | 53 |
| Nampula Central Hospital | Nampula Province | 3,985,613 | 79,010 | 50 |
| Beira Central Hospital | Sofala province | 1,642,920 | 67,753 | 24 |
